# Diagnostic Utility of Ultrasound-Guided Synovial Biopsy in Undifferentiated Monoarticular Ankle Arthritis: A Prospective Exploratory Study From Bangladesh

**DOI:** 10.7759/cureus.117778

**Published:** 2026-10-11

**Authors:** Mohammad Abul Kalam Azad, M Masudul Hassan, Daluar Hossain, Md Nahiduzzamane Shazzad, Ariful Islam, Abul Khair Ahmed Ullah, Minhaj Rahim Choudhury

**Affiliations:** 1 Rheumatology, Bangladesh Medical University, Dhaka, BGD; 2 Rheumatology, Bangladesh College of Physicians and Surgeons, Dhaka, BGD; 3 Rheumatology, Japan Bangladesh Friendship Hospital, Dhaka, BGD

**Keywords:** acid-fast bacilli, diagnostic accuracy, genexpert mtb/rif, histopathology, monoarticular ankle arthritis, synovitis, tuberculous arthritis, ultrasound-guided synovial biopsy

## Abstract

Background: Monoarticular ankle arthritis has a broad differential diagnosis, and establishing the underlying aetiology can be challenging when initial clinical, laboratory, and imaging investigations are inconclusive. Ultrasound-guided synovial biopsy enables tissue sampling for histopathological and microbiological evaluation. This study evaluated the histopathological findings and potential role of ultrasound-guided synovial biopsy in patients with undifferentiated monoarticular ankle arthritis.

Methods: This prospective, single-centre exploratory study was conducted at Bangladesh Medical University, Dhaka, Bangladesh, from January to December 2025. Thirty consecutive adults with monoarticular ankle arthritis underwent standardised clinical, laboratory, and imaging evaluation. Eight patients (26.7%) received a clinical diagnosis without biopsy, whereas 22 (73.3%) with undifferentiated arthritis underwent ultrasound-guided synovial biopsy. Within the biopsy cohort, a prespecified histopathological classification was used to classify cases as tuberculous or nontuberculous arthritis; this classification was not considered an independent microbiological reference standard. The GeneXpert Mycobacterium tuberculosis/rifampicin (MTB/RIF) assay (Cepheid, Sunnyvale, CA, USA) and acid-fast bacilli (AFB) staining were performed on available synovial tissue and fluid specimens. Sensitivity, specificity, and diagnostic accuracy were calculated with exact 95% CIs.

Results: Among the 30 participants, 22 (73.3%) had undifferentiated arthritis and underwent biopsy. Their mean age was 38.5 ± 15.0 years, and 17 of the 22 (77.3%) were male. Based on the prespecified histopathological classification, five of the 22 (22.7%) were classified as having tuberculous arthritis and 17 (77.3%) as having nontuberculous arthritis, most commonly nonspecific synovitis. Granulomatous inflammation was present in all five (100.0%) classified tuberculous cases and zero of the 17 (0%) nontuberculous cases (p < 0.001). Epithelioid cell granulomas and caseous necrosis were present in four of the five (80.0%) and three of the five (60.0%) classified tuberculous cases, respectively. Tissue GeneXpert MTB/RIF showed a sensitivity of 60.0% (3/5; 95% CI, 14.7-94.7%), specificity of 100.0% (17/17; 95% CI, 80.5-100.0%), and diagnostic accuracy of 90.9% (20/22). Synovial-fluid GeneXpert MTB/RIF showed a sensitivity of 20.0% (1/5; 95% CI, 0.5-71.6%), specificity of 100.0% (17/17; 95% CI, 80.5-100.0%), and diagnostic accuracy of 81.8% (18/22). AFB staining of tissue and synovial fluid each showed a sensitivity of 20.0% (1/5; 95% CI, 0.5-71.6%), specificity of 100.0% (17/17; 95% CI, 80.5-100.0%), and diagnostic accuracy of 81.8% (18/22).

Conclusions: Ultrasound-guided synovial biopsy may provide useful diagnostic information in selected patients with undifferentiated monoarticular ankle arthritis, particularly when tuberculosis is suspected. Histopathological examination and molecular testing of synovial tissue may complement clinical, radiological, and microbiological assessment. However, these findings should be interpreted cautiously because of the exploratory study design, small number of tuberculous cases, and absence of an independent microbiological reference standard. Larger prospective studies incorporating mycobacterial culture, molecular testing, histopathology, and longitudinal follow-up are warranted to further define the role of ultrasound-guided synovial biopsy.

## Introduction

Monoarticular ankle arthritis has a broad differential diagnosis, and distinguishing infectious, inflammatory, crystal-induced, and degenerative causes can be challenging when initial investigations are inconclusive. Clinical assessment, laboratory investigations, synovial fluid analysis, and imaging may not always establish a specific aetiology, resulting in undifferentiated monoarthritis [1,2]. Identifying the underlying cause is important because treatment and prognosis vary substantially.

Tuberculosis (TB) remains an important consideration in chronic monoarthritis in endemic settings, including Bangladesh, and musculoskeletal TB can mimic other inflammatory arthritides. Tuberculous involvement of the foot and ankle is relatively uncommon and may present with nonspecific clinical, laboratory, and radiological findings, potentially delaying diagnosis and resulting in joint destruction and functional impairment [3,4]. Acid-fast bacilli (AFB) staining and molecular testing may provide additional microbiological evidence, although their diagnostic yield can be limited by the paucibacillary nature of musculoskeletal TB [4,5]. Histopathological examination may demonstrate granulomatous inflammation, epithelioid granulomas, and caseous necrosis, findings that may support a diagnosis of tuberculous synovitis when interpreted in the appropriate clinical context [6].

Synovial tissue sampling may be considered when routine investigations fail to establish the cause of persistent monoarthritis. Ultrasound guidance enables targeted synovial sampling for histopathological and, when available, microbiological or molecular examination [7,8]. Molecular testing of synovial tissue and fluid has also been investigated in tuberculous arthritis [9]. A previous small case series from Bangladesh demonstrated the feasibility and potential diagnostic utility of ultrasound-guided synovial biopsy in selected patients with undifferentiated monoarthritis [10]. The present study was conducted as a separate prospective cohort study focusing specifically on undifferentiated monoarticular ankle arthritis and the histopathological findings obtained by ultrasound-guided synovial biopsy.

However, the histopathological findings and potential diagnostic contribution of ultrasound-guided synovial biopsy in undifferentiated monoarticular ankle arthritis have not been well characterised in Bangladesh. Therefore, this study aimed to evaluate the histopathological findings obtained by ultrasound-guided synovial biopsy in patients with undifferentiated monoarticular ankle arthritis and to describe the detection of the GeneXpert Mycobacterium tuberculosis/rifampicin (MTB/RIF) assay (Cepheid, Sunnyvale, CA, USA) in synovial tissue and fluid and AFB staining findings in relation to a prespecified histopathological classification. Diagnostic-performance analyses were considered exploratory and hypothesis-generating because the prespecified histopathological classification was not an independent microbiological reference standard.

## Materials and methods

Study design and setting

This prospective, single-centre exploratory study was conducted in the Department of Rheumatology, Bangladesh Medical University (BMU; formerly Bangabandhu Sheikh Mujib Medical University (BSMMU)), Dhaka, Bangladesh, from January to December 2025. The study was conducted in accordance with the principles of the Declaration of Helsinki and reported in accordance with the Standards for Reporting Diagnostic Accuracy Studies (STARD) guidelines, to the extent applicable to this exploratory diagnostic-performance analysis [11,12].

Study population and participant selection

Thirty consecutive adults with monoarticular ankle arthritis who met the eligibility criteria were prospectively enrolled and underwent standardised clinical, laboratory, and imaging evaluation. Eight patients received a specific clinical diagnosis based on the initial clinical, laboratory, and imaging evaluation and were managed without biopsy, whereas 22 patients with persistent undifferentiated arthritis underwent ultrasound-guided synovial biopsy.

The 22 biopsied patients constituted the biopsy cohort and were included in the exploratory diagnostic-performance analyses because histopathological assessment was available. The eight non-biopsied patients were excluded from these analyses because histopathological assessment was unavailable. Their management followed the clinical diagnostic pathway and was not based on GeneXpert MTB/RIF or AFB results.

Inclusion and exclusion criteria

Adults aged ≥18 years with clinically diagnosed monoarticular ankle arthritis presenting during the study period were eligible. Monoarticular ankle arthritis was defined as clinical evidence of active inflammation involving the ankle joint without other clinically apparent inflamed peripheral joints at presentation.

Patients were excluded from enrolment if they had polyarticular arthritis, a previously established definitive diagnosis explaining the ankle arthritis, inadequate clinical or laboratory information, or declined informed consent. Active local infection at the biopsy site or other contraindications to biopsy precluded the biopsy procedure.

Among eligible participants, ultrasound-guided synovial biopsy was performed in those with persistent undifferentiated arthritis after standardised clinical, laboratory, and imaging evaluation when tissue diagnosis was considered clinically indicated.

Sample size

As this was a prospective, single-centre exploratory study designed to evaluate the histopathological findings and potential diagnostic contribution of ultrasound-guided synovial biopsy in undifferentiated monoarticular ankle arthritis, a formal hypothesis-testing sample-size calculation was not performed. Instead, consecutive eligible patients presenting during the predefined study period from January to December 2025 were enrolled.

A total of 30 eligible patients were recruited, of whom 22 had persistent undifferentiated arthritis and subsequently underwent ultrasound-guided synovial biopsy. The diagnostic-performance analyses were considered exploratory and hypothesis-generating because the biopsy cohort was small, only five patients met the prespecified criteria for histopathologically classified tuberculous arthritis, and no independent microbiological reference standard was available. The study was therefore not designed or powered to provide definitive estimates of diagnostic sensitivity or specificity.

Clinical and laboratory evaluation

Clinical assessment included demographic characteristics, disease duration, presenting symptoms, joint examination findings, comorbidities, and relevant tuberculosis exposure history. Laboratory investigations included complete blood count, erythrocyte sedimentation rate (ESR), C-reactive protein (CRP), rheumatoid factor (RF), anti-cyclic citrullinated peptide (anti-CCP) antibody, and Mantoux testing, as clinically appropriate.

Imaging and ultrasound-guided synovial biopsy

Ankle ultrasonography was performed using a Mindray DC-80 ultrasound system (Shenzhen Mindray Bio-Medical Electronics Co., Ltd., Shenzhen, China) with a 6-14 MHz high-frequency linear transducer to assess joint effusion, synovial thickening, and other features of active synovitis. In patients with persistent undifferentiated arthritis, the biopsy site was selected according to the location and accessibility of synovial hypertrophy. Biopsy was performed using an 18-gauge Bard core biopsy needle.

Ultrasound-guided synovial biopsy procedure

The procedure was performed under aseptic conditions by an experienced operator. The target synovium was identified by real-time ultrasonography, and the safest accessible trajectory was selected. After antiseptic skin preparation and sterile draping, local anaesthesia was administered with 2% lignocaine (5-10 mL).

Under continuous ultrasound guidance, the 18-gauge Bard core biopsy needle was advanced using an in-plane technique with direct visualisation. Abnormal or hypertrophied synovium was preferentially sampled. Twelve to 16 tissue cores were obtained from each patient, with six to eight cores allocated for histopathological examination and six to eight for microbiological and molecular testing.

When available, synovial fluid was collected separately for GeneXpert MTB/RIF and AFB staining. Tissue specimens were placed in appropriately labelled containers and processed according to standard laboratory procedures.

After biopsy, local pressure was applied to achieve haemostasis and a sterile dressing was placed. Patients were observed for immediate complications, including pain and bleeding. No immediate procedure-related complications were observed.

Histopathological and microbiological assessment

Synovial tissue was examined for chronic inflammatory cell infiltration, lymphocytic infiltration, synovial hyperplasia, fibrosis, granulomatous inflammation, epithelioid cell granulomas, and caseous necrosis. GeneXpert MTB/RIF and AFB staining were performed on available tissue and synovial fluid specimens, as applicable, according to standard laboratory procedures.

Systematic mycobacterial culture was not performed as part of the study protocol. Therefore, an independent microbiological reference standard was not available for the exploratory diagnostic-performance analyses.

Histopathological case classification

Biopsy specimens were classified into two prespecified categories: histopathologically classified tuberculous arthritis and nontuberculous/nonspecific synovitis. Histopathologically classified tuberculous arthritis was defined by the presence of granulomatous inflammation, with or without epithelioid cell granulomas and/or caseous necrosis. Specimens without granulomatous inflammation were classified as nontuberculous/nonspecific synovitis for the purposes of the prespecified exploratory analysis; this classification did not constitute definitive exclusion of TB.

This prespecified histopathological case classification was used as the reference classification for the exploratory diagnostic-performance analyses. It was not considered a microbiological gold standard and was not sufficient, in isolation, to establish microbiologically confirmed TB. Because granulomatous inflammation formed part of the classification definition, it was not assessed as an independent diagnostic test against the same classification.

Outcomes

The primary outcome was the prespecified histopathological case classification of biopsy specimens as histopathologically classified tuberculous arthritis or nontuberculous/nonspecific synovitis.

Secondary outcomes included individual histopathological findings and the exploratory diagnostic performance of GeneXpert MTB/RIF and AFB staining performed on available tissue and synovial fluid specimens.

Statistical analysis

Continuous variables were summarised as mean ± standard deviation (SD) or median with interquartile range (IQR), as appropriate, and categorical variables as frequencies and percentages. Fisher's exact test was used for categorical comparisons.

Among the 22 biopsied patients, sensitivity, specificity, positive predictive value (PPV), negative predictive value (NPV), diagnostic accuracy, and likelihood ratios (LRs) were calculated against the prespecified histopathological case classification. Exact 95% confidence intervals (CIs) for sensitivity and specificity were calculated using the Clopper-Pearson method.

When no false-positive results occurred, specificity was 100% and the positive likelihood ratio (LR+) was considered infinite. These estimates were interpreted cautiously because of the small number of participants and the absence of an independent microbiological reference standard.

Diagnostic-performance estimates were considered exploratory and hypothesis-generating because only five patients met the prespecified criteria for histopathologically classified tuberculous arthritis. They were not interpreted as definitive estimates of the diagnostic accuracy of GeneXpert MTB/RIF for tuberculous arthritis.

All analyses were performed using IBM SPSS Statistics, version 27 (IBM Corp., Armonk, NY, USA). A two-sided p < 0.05 was considered statistically significant; no adjustment for multiple comparisons was performed.

Follow-up and interpretation of the nontuberculous/nonspecific group

Long-term systematic follow-up was not part of the study protocol. Accordingly, classification of patients as nontuberculous/nonspecific synovitis was based on the prespecified histopathological criteria and should not be interpreted as definitive exclusion of tuberculosis or other specific infectious or inflammatory diagnoses. This limitation was considered when interpreting the exploratory diagnostic-performance estimates.

Ethical considerations

The Institutional Review Board of Bangladesh Medical University approved the study (IRB No. BSMMU/2024/10711). Written informed consent was obtained from all participants before enrolment, and additional consent for the biopsy procedure was obtained before biopsy

## Results

Participant characteristics and initial clinical classification

A total of 30 adults with monoarticular ankle arthritis were assessed. Eight (8/30, 26.7%) received a specific clinical diagnosis and were managed without biopsy, whereas 22 (22/30, 73.3%) had undifferentiated arthritis and underwent ultrasound-guided synovial biopsy. All biopsy specimens were adequate for histopathological evaluation, and synovial fluid was available for microbiological and molecular testing in all 22 patients. Initial clinical diagnoses included gouty arthritis (3/30, 10.0%), spondyloarthritis (2/30, 6.7%), psoriatic arthritis (2/30, 6.7%), and rheumatoid arthritis (1/30, 3.3%) (Table 1).

**Table 1:** Initial Clinical Classification of the Study Participants (n = 30) Values are presented as n (%). The clinical-diagnosis group comprised patients with a specific diagnosis based on the initial clinical, laboratory, and imaging evaluation who were managed without synovial biopsy. Undifferentiated ankle arthritis refers to patients in whom a definitive diagnosis was not established during the initial evaluation and who subsequently underwent ultrasound-guided synovial biopsy.

Clinical diagnosis or classification	n (%)
Clinical diagnosis established without biopsy	8 (26.7)
Gouty arthritis	3 (10.0)
Spondyloarthritis	2 (6.7)
Psoriatic arthritis	2 (6.7)
Rheumatoid arthritis	1 (3.3)
Undifferentiated ankle arthritis	22 (73.3)
Total	30 (100.0)

Baseline sociodemographic and clinical characteristics

Among the 22 biopsy patients, the mean age was 38.5 ± 15.0 years (range, 20-80), and 17 (17/22, 77.3%) were male. Garment workers (7/22, 31.8%) and cultivators (5/22, 22.7%) were the largest occupational groups. Low, middle, and high socioeconomic status were present in 11 (50.0%), eight (36.4%), and three (13.6%) of these 22 patients, respectively.

Fourteen patients (14/22, 63.6%) had no comorbidity; diabetes mellitus and hypertension were present in seven patients (7/22, 31.8%) and one patient (1/22, 4.5%), respectively. Chronic arthritis was present in 21 patients (21/22, 95.5%). Right- and left-ankle involvement was equal (11/22, 50.0% each). Symptom duration was six months or less in 12 patients (12/22, 54.5%) and more than six months in 10 patients (10/22, 45.5%) (Table 2).

**Table 2:** Baseline Sociodemographic and Clinical Characteristics of the Biopsy Cohort (n = 22) Values are presented as n (%) unless otherwise indicated. SD, standard deviation.

Variable	Category	Value
Age (years)	Mean ± SD	38.5 ± 15.0
Age (years)	Range	20-80
Sex	Male	17 (77.3)
	Female	5 (22.7)
Occupation	Garment worker	7 (31.8)
	Cultivator	5 (22.7)
	Student	4 (18.2)
	Homemaker	4 (18.2)
	Other	2 (9.1)
Socioeconomic status	Low	11 (50.0)
	Middle	8 (36.4)
	High	3 (13.6)
Comorbidity	None	14 (63.6)
	Diabetes mellitus	7 (31.8)
	Hypertension	1 (4.5)
Disease course	Chronic arthritis	21 (95.5)
	Acute/subacute arthritis	1 (4.5)
Ankle involvement	Right	11 (50.0)
	Left	11 (50.0)
Symptom duration	≤6 months	12 (54.5)
	>6 months	10 (45.5)

Laboratory and imaging findings

Mean ESR was 69.8 ± 29.7 mm/h. CRP was elevated in 14 patients (14/22, 63.6%), while RF and anti-CCP antibodies were negative in all 22 patients (22/22, 100.0%). Mantoux testing was positive in 11 patients (11/22, 50.0%). Ultrasonography showed soft-tissue swelling in 14 patients (14/22, 63.6%), joint effusion in nine patients (9/22, 40.9%), and synovial thickening in eight patients (8/22, 36.4%) (Table 3).

**Table 3:** Laboratory and Ultrasonographic Findings in the Biopsy Cohort (n = 22) Values are presented as n (%) unless otherwise indicated. Anti-CCP, anti-cyclic citrullinated peptide antibody; CRP, C-reactive protein; ESR, erythrocyte sedimentation rate; RF, rheumatoid factor; SD, standard deviation.

Parameter	Result
ESR (mm/h), mean ± SD	69.8 ± 29.7
Elevated C-reactive protein	14 (63.6)
RF negative	22 (100.0)
Anti-CCP negative	22 (100.0)
Mantoux test positive	11 (50.0)
Soft-tissue swelling on ultrasonography	14 (63.6)
Joint effusion on ultrasonography	9 (40.9)
Synovial thickening on ultrasonography	8 (36.4)

Histopathological findings

Chronic inflammatory cell infiltration was the most frequent histopathological finding (18/22, 81.8%), followed by nonspecific synovitis (17/22, 77.3%), lymphocytic infiltration (16/22, 72.7%), synovial hyperplasia (10/22, 45.5%), and fibrosis (6/22, 27.3%). Granulomatous inflammation, epithelioid cell granuloma, and caseous necrosis were present in five patients (5/22, 22.7%), four patients (4/22, 18.2%), and three patients (3/22, 13.6%), respectively (Table 4).

**Table 4:** Histopathological Findings of Synovial Biopsy Specimens in the Biopsy Cohort (n = 22) Values are presented as n (%). Histopathological findings were not mutually exclusive; therefore, percentages do not sum to 100%. All five patients with granulomatous inflammation were classified as having histopathologically classified tuberculous arthritis according to the prespecified classification.

Histopathological feature	n (%)
Chronic inflammatory cell infiltration	18 (81.8)
Lymphocytic infiltration	16 (72.7)
Nonspecific synovitis	17 (77.3)
Synovial hyperplasia	10 (45.5)
Fibrosis	6 (27.3)
Granulomatous inflammation	5 (22.7)
Epithelioid cell granuloma	4 (18.2)
Caseous necrosis	3 (13.6)

Histopathological classification

According to the prespecified classification, five patients (5/22, 22.7%) had histopathologically classified tuberculous arthritis and 17 patients (17/22, 77.3%) had nontuberculous/nonspecific synovitis.

Granulomatous inflammation was present in five patients (5/5, 100.0%) in the histopathologically classified tuberculous group and zero patients (0/17, 0.0%) in the nontuberculous/nonspecific group. Epithelioid cell granuloma was present in four patients (4/5, 80.0%) and zero patients (0/17, 0.0%), respectively, while caseous necrosis was present in three patients (3/5, 60.0%) and zero patients (0/17, 0.0%), respectively. Nonspecific synovitis was present in 17 patients (17/17, 100.0%) in the nontuberculous/nonspecific group and was absent in the histopathologically classified tuberculous group. Chronic inflammatory cell infiltration was present in five patients (5/5, 100.0%) versus 13 patients (13/17, 76.5%), respectively (p = 0.535) (Table 5).

**Table 5:** Distribution of Histopathological Features According to the Prespecified Classification in the Biopsy Cohort (n = 22) Values are presented as n (%). The groups were defined according to the prespecified histopathological classification and should not be interpreted as independent microbiological reference-standard classifications. Because granulomatous inflammation was incorporated into the classification definition, and the other related histopathological features contributed to the classification, these findings are presented descriptively and were not subjected to independent significance testing.

Histopathological feature	Histopathologically classified as tuberculous, n = 5	Nontuberculous/nonspecific, n = 17	p-value
Chronic inflammatory cell infiltration	5 (100.0)	13 (76.5)	0.535
Granulomatous inflammation	5 (100.0)	0 (0.0)	-
Epithelioid cell granuloma	4 (80.0)	0 (0.0)	-
Caseous necrosis	3 (60.0)	0 (0.0)	-
Nonspecific synovitis	0 (0.0)	17 (100.0)	-

Exploratory diagnostic performance of GeneXpert MTB/RIF and AFB staining

Tissue GeneXpert MTB/RIF was positive in three cases (3/5, 60.0%) of the histopathologically classified tuberculous group and negative in 17 cases (17/17, 100.0%) of the nontuberculous/nonspecific group. Sensitivity was 60.0% (3/5; 95% CI, 14.7-94.7%), specificity was 100.0% (17/17; 95% CI, 80.5-100.0%), PPV was 100.0% (3/3), NPV was 89.5% (17/19), and diagnostic accuracy was 90.9% (20/22). LR+ was infinite and the negative likelihood ratio (LR-) was 0.40.

Synovial-fluid GeneXpert MTB/RIF was positive in one case (1/5, 20.0%) of the histopathologically classified tuberculous group and negative in 17 cases (17/17, 100.0%) of the nontuberculous/nonspecific group. Sensitivity was 20.0% (1/5; 95% CI, 0.5-71.6%), specificity was 100.0% (17/17; 95% CI, 80.5-100.0%), PPV was 100.0% (1/1), NPV was 81.0% (17/21), and diagnostic accuracy was 81.8% (18/22). LR+ was infinite and LR- was 0.80.

Tissue and synovial-fluid AFB staining each showed positivity in one case (1/5, 20.0%) of the histopathologically classified tuberculous group and no positive results among the 17 cases (0/17, 0.0%) of the nontuberculous/nonspecific group. For both tests, sensitivity was 20.0% (1/5; 95% CI, 0.5-71.6%), specificity was 100.0% (17/17; 95% CI, 80.5-100.0%), PPV was 100.0% (1/1), NPV was 81.0% (17/21), and diagnostic accuracy was 81.8% (18/22); LR+ was infinite and LR- was 0.80 (Table 6).

**Table 6:** Exploratory Diagnostic Performance of GeneXpert MTB/RIF and AFB Staining Relative to the Prespecified Histopathological Classification in the Biopsy Cohort (n = 22) AFB, acid-fast bacilli; CI, confidence interval; FN, false negative; FP, false positive; LR-, negative likelihood ratio; LR+, positive likelihood ratio; MTB/RIF, Mycobacterium tuberculosis/rifampicin; NPV, negative predictive value; PPV, positive predictive value; TN, true negative; TP, true positive. The GeneXpert MTB/RIF assay is manufactured by Cepheid (Sunnyvale, CA, USA). Diagnostic-performance estimates were calculated relative to the prespecified histopathological classification among the 22 patients in the biopsy cohort, of whom five were classified as having histopathologically classified tuberculous arthritis and 17 as nontuberculous/nonspecific synovitis. Exact 95% CIs were calculated using the Clopper-Pearson method. No false-positive results were observed; therefore, the observed specificity was 100.0% and LR+ was infinite for all tests. These estimates should be interpreted cautiously because of the small number of histopathologically classified tuberculous cases and the lack of an independent microbiological reference standard.

Diagnostic test	TP	FN	TN	FP	Sensitivity, % (95% CI)	Specificity, % (95% CI)	PPV, %	NPV, %	Accuracy, %	LR+	LR-
Tissue GeneXpert MTB/RIF	3	2	17	0	60.0 (14.7-94.7)	100.0 (80.5-100.0)	100.0	89.5	90.9	∞	0.40
Synovial-fluid GeneXpert MTB/RIF	1	4	17	0	20.0 (0.5-71.6)	100.0 (80.5-100.0)	100.0	81.0	81.8	∞	0.80
Tissue AFB staining	1	4	17	0	20.0 (0.5-71.6)	100.0 (80.5-100.0)	100.0	81.0	81.8	∞	0.80
Synovial-fluid AFB staining	1	4	17	0	20.0 (0.5-71.6)	100.0 (80.5-100.0)	100.0	81.0	81.8	∞	0.80

Tissue GeneXpert MTB/RIF had the highest observed sensitivity (3/5, 60.0%) and diagnostic accuracy (20/22, 90.9%). No false-positive results were observed among the 17 patients classified as nontuberculous/nonspecific; therefore, the observed specificity was 100.0% for all evaluated tests. However, these estimates were based on only 22 biopsied patients, of whom five met the prespecified histopathological criteria for histopathologically classified tuberculous arthritis and 17 were classified as nontuberculous/nonspecific synovitis. Therefore, the diagnostic-performance estimates should be interpreted as exploratory and hypothesis-generating rather than definitive measures of diagnostic accuracy.

## Discussion

Diagnosing monoarticular ankle arthritis can be challenging and requires integration of clinical assessment, imaging, microbiological investigations, and, when indicated, histopathological examination [1,2]. In the present study, a clinical diagnosis was established without biopsy in eight of 30 patients (26.7%), whereas the remaining 22 (73.3%) had persistent undifferentiated arthritis after initial evaluation and underwent ultrasound-guided synovial biopsy. These findings support the potential role of synovial biopsy in selected patients with persistent undifferentiated monoarthritis [2,10].

Among the 22 patients who underwent biopsy, the mean age was 38.5 ± 15.0 years, and 77.3% were male. Most patients had chronic arthritis, and inflammatory markers were frequently elevated. The Mantoux test was positive in 50.0%. However, a positive tuberculin skin test alone cannot establish active tuberculosis and should be interpreted in conjunction with clinical, radiological, histopathological, and microbiological findings [3,4].

Based on the prespecified histopathological criteria, five of the 22 biopsied patients (22.7%) met the criteria for histopathologically classified tuberculous arthritis. Epithelioid cell granulomas were present in four patients (80.0%), and caseous necrosis in three (60.0%). These findings are consistent with recognised histopathological features of osteoarticular tuberculosis [6,13]. A previous retrospective study reported clinical and diagnostic characteristics of osteoarticular tuberculosis [14]. Granulomatous inflammation represents an important component of the host response to tuberculosis [15]. Although caseous necrosis may support the diagnosis, its absence does not exclude tuberculosis [6,13]. Because granulomatous inflammation was incorporated into the prespecified classification criteria, its presence should not be interpreted as an independent measure of diagnostic performance.

Tissue GeneXpert MTB/RIF was positive in three of the five patients classified by the prespecified histopathological criteria as tuberculous (60.0%), whereas synovial-fluid GeneXpert was positive in one case (20.0%). Thus, tissue GeneXpert showed a higher observed positivity rate in this cohort. Previous studies have reported variable diagnostic performance of molecular assays for osteoarticular tuberculosis, with results influenced by specimen type and disease characteristics [5,9,13,16]. However, the small number of histopathologically classified tuberculous cases limits the precision of these observations. Therefore, this difference should be considered exploratory and should not be interpreted as evidence of superior diagnostic performance of tissue GeneXpert.

AFB staining was positive in only one of the five patients classified by the prespecified histopathological criteria as tuberculous (20.0%), consistent with the limited yield of direct microscopic detection in osteoarticular tuberculosis [5,13]. Although no false-positive results were observed and the observed specificity was 100% for the evaluated tests, this estimate should not be interpreted as evidence of perfect specificity because only 17 patients were classified as nontuberculous/nonspecific and no independent microbiological reference standard was available.

Nonspecific synovial inflammation was observed in 17 of the 22 biopsy specimens (77.3%). This finding indicates that synovial histopathology may remain nondiagnostic in a substantial proportion of patients with undifferentiated monoarthritis. Therefore, biopsy findings should be interpreted together with the clinical presentation, imaging findings, and microbiological investigations [2,7]. The absence of specific histopathological findings should not be regarded as definitive exclusion of tuberculosis or other specific infectious or inflammatory disorders.

A strength of this study is its prospective evaluation of ultrasound-guided synovial biopsy in patients with undifferentiated monoarticular ankle arthritis in a tuberculosis-endemic setting. Ultrasound guidance permits targeted sampling of abnormal synovium and provides tissue for histopathological and, when available, microbiological and molecular investigations [7,8]. The study also evaluated GeneXpert MTB/RIF and AFB staining in available synovial tissue and fluid specimens. The higher observed positivity of tissue GeneXpert suggests that tissue sampling may provide additional diagnostic information when tuberculosis is suspected; however, this observation requires confirmation in larger, adequately powered studies using an independent reference standard.

Limitations

This study has several limitations. First, it was a single-centre study involving only 30 patients, of whom 22 underwent biopsy. Second, only five patients met the prespecified histopathological criteria for histopathologically classified tuberculous arthritis, limiting the precision of the diagnostic estimates and precluding robust comparisons between diagnostic modalities. Third, histopathological assessment was performed only in patients who underwent biopsy, which may have introduced selection and verification bias and limits the generalisability of the findings. Fourth, no independent microbiological reference standard was available; systematic mycobacterial culture was not performed, and microbiological testing therefore could not independently validate the histopathological classification. Consequently, both microbiologically unconfirmed tuberculosis and misclassification remain possible [5,13]. Fifth, mycobacterial culture, immunohistochemistry, and additional molecular assays were not included in the study protocol and might have provided additional diagnostic evidence. Finally, long-term systematic follow-up was not part of the study protocol, limiting assessment of the subsequent clinical course and final diagnosis among patients classified as having nontuberculous/nonspecific synovitis.

## Conclusions

Ultrasound-guided synovial biopsy may provide useful diagnostic information in selected patients with persistent undifferentiated monoarticular ankle arthritis, particularly when TB is suspected. Histopathological examination and molecular testing, including GeneXpert MTB/RIF, may complement clinical, radiological, and microbiological assessment. However, the findings should be interpreted cautiously because of the exploratory study design, small number of histopathologically classified tuberculous cases, potential selection and verification bias, and absence of an independent microbiological reference standard. Larger prospective studies incorporating systematic mycobacterial culture, molecular testing, histopathology, and longitudinal follow-up are warranted to further define the diagnostic contribution of ultrasound-guided synovial biopsy.

## References

[1] Till SH, Snaith ML (1999). Assessment, investigation, and management of acute monoarthritis. J Accid Emerg Med.

[2] Soroosh SG, Ghatfan A, Farbod A, Meftah E (2023). Synovial biopsy for establishing a definite diagnosis in undifferentiated chronic knee monoarthritis. BMC Musculoskelet Disord.

[3] Faroug R, Psyllakis P, Gulati A, Makvana S, Pareek M, Mangwani J (2018). Diagnosis and treatment of tuberculosis of the foot and ankle-a literature review. Foot (Edinb).

[4] Zhou J, Yang X, Hu Y, Li S (2025). Epidemiological and osteoarticular involvement sites' characteristics of multiple osteoarticular tuberculosis: a scoping review. Epidemiol Infect.

[5] Negi SS, Gupta S, Khare S, Lal S (2005). Comparison of various microbiological tests including polymerase chain reaction for the diagnosis of osteoarticular tuberculosis. Indian J Med Microbiol.

[6] Sant M, Bajaj H (1992). Role of histopathology in the diagnosis of tuberculous synovitis. J Indian Med Assoc.

[7] Sitt JC, Griffith JF, Wong P (2016). Ultrasound-guided synovial biopsy. Br J Radiol.

[8] Sitt JC, Griffith JF, Lai FM (2017). Ultrasound-guided synovial Tru-cut biopsy: indications, technique, and outcome in 111 cases. Eur Radiol.

[9] Titov AG, Vyshnevskaya EB, Mazurenko SI, Santavirta S, Konttinen YT (2004). Use of polymerase chain reaction to diagnose tuberculous arthritis from joint tissues and synovial fluid. Arch Pathol Lab Med.

[10] Azad MA, Hassan MM, Choudhury MR, Islam A, Ahmedullah AK (2025). Ultrasound-guided synovial biopsy in undifferentiated monoarthritis: a case series from Bangladesh. Cureus.

[11] (2013). World Medical Association Declaration of Helsinki: ethical principles for medical research involving human subjects. JAMA.

[12] Bossuyt PM, Reitsma JB, Bruns DE (2015). STARD 2015: an updated list of essential items for reporting diagnostic accuracy studies. BMJ.

[13] Abhimanyu S, Jain AK, Myneedu VP, Arora VK, Chadha M, Sarin R (2021). The role of cartridge-based nucleic acid amplification test (CBNAAT), line probe assay (LPA), liquid culture, acid-fast bacilli (AFB) smear and histopathology in the diagnosis of osteoarticular tuberculosis. Indian J Orthop.

[14] Arathi N, Ahmad F, Huda N (2013). Osteoarticular tuberculosis-a three years' retrospective study. J Clin Diagn Res.

[15] Lyu J, Narum DE, Baldwin SL (2024). Understanding the development of tuberculous granulomas: insights into host protection and pathogenesis, a review in humans and animals. Front Immunol.

[16] Gong X, He Y, Zhou K, Hua Y, Li Y (2023). Efficacy of Xpert in tuberculosis diagnosis based on various specimens: a systematic review and meta-analysis. Front Cell Infect Microbiol.

